# Efficacy and safety of USG-guided ethanol sclerotherapy in cystic thyroid nodules

**DOI:** 10.4103/0971-3026.54879

**Published:** 2009-08

**Authors:** SR Jayesh, Pankaj Mehta, Mathew P Cherian, V Ilayaraja, Prashanth Gupta, K Venkatesh

**Affiliations:** Department of Radiology, Kovai Medical Center and Hospital, Avinashi Road, Coimbatore, Tamil Nadu, India

**Keywords:** Cystic thyroid nodules, ethanol sclerotherapy

## Abstract

**Objective:**

To study the effectiveness and safety of USG-guided ethanol sclerotherapy in cystic thyroid nodules.

**Materials and Methods:**

USG of the thyroid gland was performed in 54 patients suspected to have a thyroid nodule on clinical examination. All patients with a predominantly cystic nodule (i.e., when >2/3^rd^ of the nodule was cystic) were included in the study. Ethanol was injected into the cyst under USG guidance. The amount of ethanol injected was about 50% of the amount of aspirated fluid. Follow-up USG was done every month for 3 months; ethanol was re-injected when there was no significant reduction in the cyst volume. The initial cyst volume was compared with the final volume; statistical significance was assessed using the paired *t*-test.

**Results:**

USG revealed predominant cystic nodules in 16 of the 54 patients. Fifteen patients were selected for the study. Following ethanol sclerotherapy, four out of the 15 patients (26.6%) showed complete disappearance of the cyst and nine (60%) showed significant reduction in the cyst volume (i.e., reduction of cyst volume by ≥50% of initial volume). Only two patients did not show significant reduction in cyst volume; both these patients had nodules with an initial volume of ≥20 cc. There were no complications attributable to ethanol injection during follow-up.

**Conclusion:**

Ethanol sclerotherapy is an effective and safe treatment for benign cystic thyroid nodules with volumes of <20 cc. Cystic nodules with volume >20 cc may need more number of alcohol injections and longer follow-up.

## Introduction

Thyroid disease can present as solitary or multiple nodules in symptomatic or asymptomatic patients. The contents of a thyroid nodule can be solid or cystic in various proportions. According to various studies, 15–30% of thyroid nodules are cystic or predominantly cystic.[1–5] Most of the malignancies are seen in solid and hypoechoic nodules, but the presence of a cystic nodule does not rule out malignancy.[3,5] Studies have shown that benign cystic thyroid nodules treated by simple aspiration show high rates of recurrence.[6,7] Surgical treatment of a cystic thyroid nodule is curative although there is a risk of complications, e.g., general anesthesia-related complications, postoperative scar formation and hypothyroidism. According to the American Thyroid Association and the European Thyroid Association, a conservative (or nonsurgical) approach should be used for benign cystic thyroid nodules. Treatment of benign cystic thyroid nodules is generally undertaken for cosmetic reasons or for local compressive manifestations such as dysphagia.[8]

Radioactive iodine ablation, a nonsurgical method of treatment, is effective but requires special equipment.[9] Another option includes tetracycline instillation; however, this method has not been proven to be capable of producing any statistically significant reduction in the size of thyroid cysts.[10]

Sclerotherapy using percutaneous ethanol injection (PEI) is another nonsurgical procedure that has drawn attention as a therapeutic option in cystic thyroid nodules.[11] The aim of this study was to evaluate the effectiveness and safety of USG-guided PEI in cystic thyroid nodules.

## Materials and Methods

This prospective study was conducted over a period of one year. Fifty-four patients clinically suspected to have a thyroid nodule were subjected to USG of the thyroid gland. USG was performed using one of the three scanners (Voluson 730 pro, Voluson I and Logic E, GE Medical Systems, Bangalore, India), using a 5–10 MHz linear probe. In patients with predominantly cystic thyroid nodules (i.e., >2/3rd the cystic component), fine-needle aspiration cytology (FNAC) was performed under strict aseptic precautions, using 21–22 G hypodermic needles. Septate cysts have higher recurrence rates and were not included in this study because the distribution and spread of ethanol within these cysts is poor. Patients with FNAC-proven benign cystic lesions underwent ethanol sclerotherapy mainly for their cosmetic complaints; informed consent was obtained in all cases. There were 16 patients with benign cystic lesions. One patient was only 13 years old and was considered too young to be included in the study; 15 patients underwent ethanol sclerotherapy. The initial cyst volume was calculated using machine-generated values from the transverse and longitudinal dimensions.

**Figure 1:**
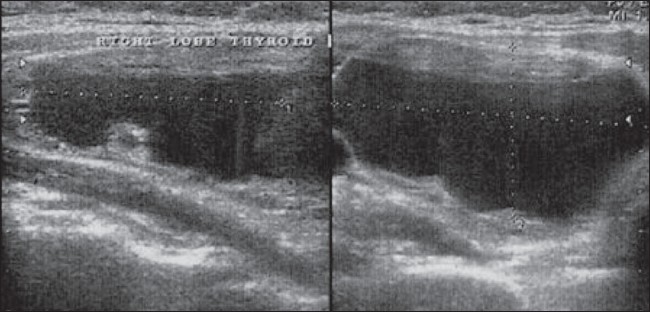
Longitudinal USG shows a cystic thyroid nodule before ethanol injection

**Figure 2:**
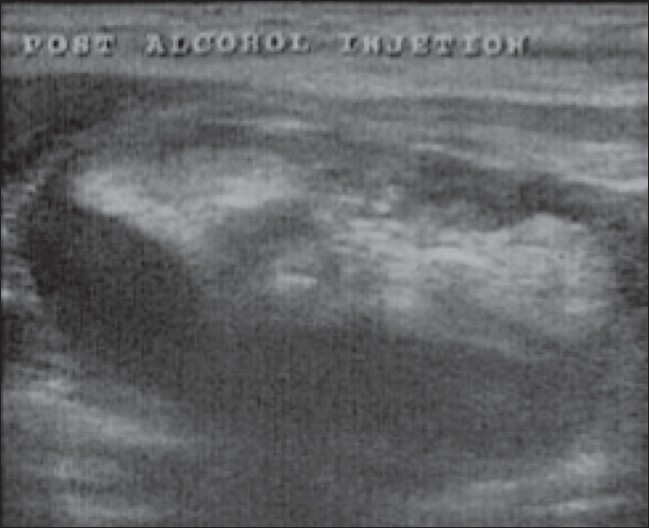
Longitudinal USG shows a hyperechoic ethanol component immediately after ethanol injection

The cyst was first aspirated; 95% ethanol was then slowly injected into the cyst with USG guidance. Local anesthesia was not used. The amount of alcohol injected was about 50% of the amount of fluid aspirated. Gentle pressure was applied over the puncture site for 10–15 min and the patient was watched for signs of any complications [Figures 1 and 2].

**Figure 3:**
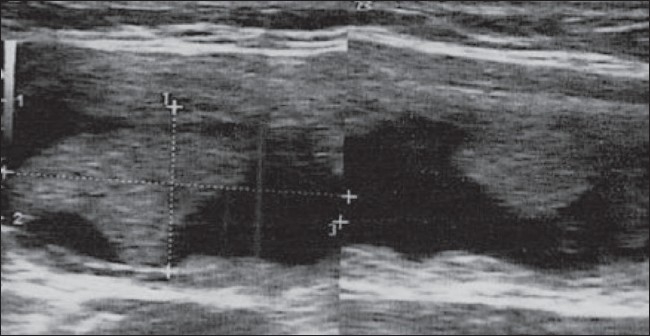
Longitudinal USG shows some persistence of a cystic component at the first follow-up visit

**Figure 4:**
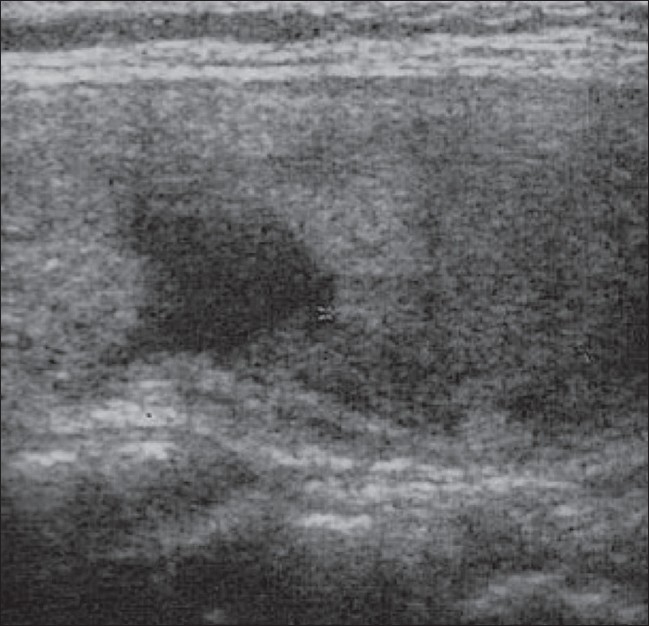
Longitudinal USG shows significant reduction of the cystic component at the end of the third month

All patients were followed every month for up to 3 months. A USG scan was performed at every follow-up visit to assess the cyst volume. Alcohol was re-injected if there was no significant reduction in the cyst volume (i.e., reduction by ≥50% of the initial volume) [Figures 3 and 4]. At each follow-up, patients were evaluated for the presence of side effects such as pain, burning sensation or respiratory discomfort.

**Figure 5:**
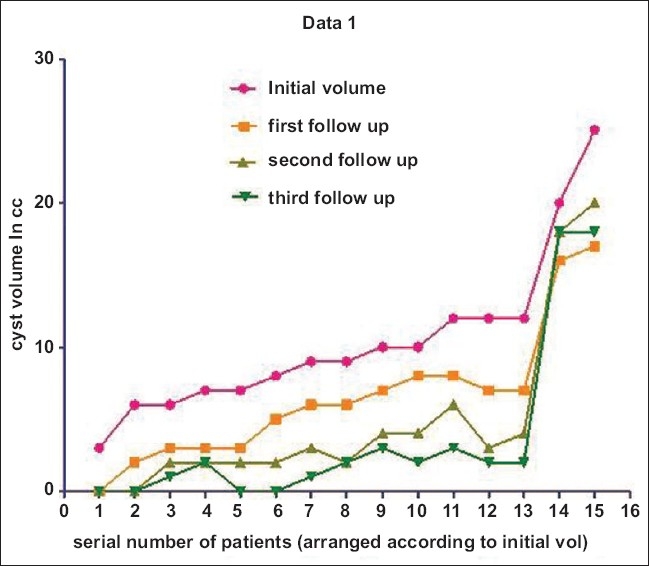
The graph shows the mean cyst volume at monthly followup up to 3 months

## Results

The mean initial cyst volume before sclerotherapy was 10.4 cc (range: 3–25 cc), and the mean amount of alcohol injected at the first sitting was 4.8 ml (range: 1.5–10 ml). At the first follow-up, 5 out of 15 patients showed significant reduction in cyst volume (i.e., ≥50% of the initial volume).[Table 1] The 10 remaining patients underwent a second sitting of alcohol injection. The mean cyst volume at the second sitting was 8.7 cc (range: 5–17 cc) and the mean amount of alcohol injected was 4 ml (range: 2–8 ml). At the second follow-up visit, 8 out of 10 patients who underwent re-injection showed significant reduction of cyst volume. Two patients with initial volumes of ≥20 cc showed no significant reduction even after a third sitting [Table 1]. At the end of the third follow-up, 13 of the 15 cases (86.6%) showed a significant reduction in cyst volumes. Four out of 15 (26.6%) patients showed complete disappearance of the cyst [Figure 5]. The paired *t*-test was used to evaluate the significance of the difference between the initial volume (before ethanol sclerotherapy) and the final volume (cyst volume after ethanol injection). There was statistically significant reduction in the cyst volume (*P* < 0.001) after ethanol injection at the end of the 3-month follow-up.

**Table 1:** Percentage reduction of cystic thyroid nodule volume after ethanol sclerotherapy

Serial No.	Initial volume in cc	1^st^ f/u volume in cc	Percentage* reduction	2^nd^ f/u volume in cc	Percentage* reduction	Final volume in cc	Percentage* reduction
1	3	0	Complete	0	Complete	0	Complete
2	6	2	>50	0	Complete	0	Complete
3	6	3	50	2	>50	1	>50
4	7	3	>50	2	>50	2	>50
5	7	3	>50	2	>50	0	Complete
6	8	5	<50	2	>50	0	Complete
7	9	6	<50	3	>50	1	>50
8	9	6	<50	2	>50	2	>50
9	10	7	<50	4	>50	3	>50
10	10	8	>50	4	>50	2	>50
11	12	8	<50	6	>50	3	>50
12	12	7	<50	3	>50	2	>50
13	12	7	<50	4	>50	2	>50
14	20	16	<50	18	<50	18	<50
15	25	17	<50	20	<50	18	<50

* Percentage reduction in comparison to the initial volume. Numbers in green - first sitting alcohol injection. Numbers in blue - second sitting alcohol injection. Numbers in red - third sitting alcohol injection

There were no complications following ethanol injection or during follow-up.

## Discussion

Patients with benign cystic thyroid nodules usually seek treatment for cosmetic reasons or because of local compressive manifestations, such as dysphagia.[8] Simple cyst aspiration can decrease the size, but the recurrence rate is in the range of 10–80% depending on the cyst volume.[6,7] Surgery is curative but has disadvantages, such as general anesthesia-related complications, scar formation and hypothyroidism. The use of tetracycline injections does not show significant results and radioactive iodine requires special equipment.[10]

Treatment of thyroid cysts with PEI was first proposed in 1987 by Edmonds *et al*[12] and later in 1989 by Rozman *et al*.[13] The proposed mechanism of action is as follows: after injection of ethanol into the nodule, there is epithelial cell dehydration and protein denaturation. This is followed by coagulative necrosis, reactive fibrosis and small vessel thrombosis, which result in the obliteration of the cyst.

USG-guided PEI was first proposed by Livraghi *et al*,[11] in 1990. After documenting their initial successes, they evaluated the long-term efficacy of this treatment modality and reported that patients who had had complete and partial cure did not show any recurrence during a follow-up period of 6 months to 4 years.

Verde *et al*,[14] showed that nodule volume reduction was significantly greater and more significant in patients treated with ethanol injection than in patients who had treatment with just aspiration.

In a randomized study, Bennedbaek and Laszlo Hegedus[15] showed an overall success rate of 82%, with 64% patients showing significant reduction in cyst volume after the first treatment. According to their experience and other published studies on ethanol sclerotherapy, success is defined as near-disappearance or marked size reduction (>50%) of cystic lesion. In our study, 13 out of 15 patients (86.6%) showed complete disappearance or >50% reduction in cyst volume at the end of 3 months' follow-up. Five out of 15 patients (33.3%) in our study showed a significant reduction in cyst volume after the first treatment.

In a study by Tarantino *et al*,[16] there were 12 cases with cyst volume greater than 30 ml and all these cases showed significant reduction of volume at the end of 6–9 months. In another study by Zingrillo *et al*,[17] nodules with mean volume greater than 38 ml showed significant reduction in size after 2 years. In our study, however, 2 cases (out of 15) that had initial volumes of ≥20 cc did not show any significant reduction in size at the end of 3 months' follow-up. This divergent finding may be because of the small sample size and the shorter follow-up period. Cystic nodules with volumes greater than 20 cc may need more number of alcohol injections and longer follow-ups.

### Complications associated with ethanol sclerotherapy

As with any procedure, ethanol sclerotherapy is also associated with some complications. According to literature, mild transient pain or a burning sensation at the site of injection are the most commonly seen side effects following ethanol sclerotherapy and are as a result of leakage of ethanol into the subcutaneous tissue. Other uncommon complications include hematoma, dyspnea and vocal cord paralysis. Lee and Ahn,[9] in 2005 carried out ethanol injections in 432 patients, with long-term follow-up. Only 9% of their patients showed complications like mild pain at the injection site. There were no permanent complications or side effects. Our patients did not show any complications or side effects.

In conclusion, ethanol sclerotherapy is an effective and safe nonsurgical treatment option for benign cystic thyroid nodules with volumes of less than 20 cc. Cystic nodules with volumes greater than 20 cc may need more number of alcohol injections and longer follow-up before results become evident.
